# Specific Sialoforms Required for the Immune Suppressive Activity of Human Soluble CD52

**DOI:** 10.3389/fimmu.2019.01967

**Published:** 2019-08-27

**Authors:** Abdulrahman M. Shathili, Esther Bandala-Sanchez, Alan John, Ethan D. Goddard-Borger, Morten Thaysen-Andersen, Arun V. Everest-Dass, Timothy E. Adams, Leonard C. Harrison, Nicolle H. Packer

**Affiliations:** ^1^Department of Molecular Sciences, Macquarie University, Sydney, NSW, Australia; ^2^ARC Centre of Nanoscale Biophotonics, Macquarie University, Sydney, NSW, Australia; ^3^Al-Rayan Research and Innovation Centre, Alrayan Medical Colleges, Madinah, Saudi Arabia; ^4^The Walter and Eliza Hall Institute of Medical Research, Parkville, VIC, Australia; ^5^Department of Medical Biology, University of Melbourne, Parkville, VIC, Australia; ^6^Institute for Glycomics, Griffith University, Brisbane, QLD, Australia; ^7^Manufacturing (CSIRO), Parkville, VIC, Australia

**Keywords:** CD52, immune suppression, glycan structure, analysis, tetra-antennary, α-2,3 sialylation

## Abstract

Human CD52 is a small glycopeptide (12 amino acid residues) with one *N-*linked glycosylation site at asparagine 3 (Asn3) and several potential *O-*glycosylation serine/threonine sites. Soluble CD52 is released from the surface of activated T cells and mediates immune suppression via its glycan moiety. In suppressing activated T cells, it first sequesters the pro-inflammatory high mobility group Box 1 (HMGB1) protein, which facilitates its binding to the inhibitory sialic acid-binding immunoglobulin-like lectin-10 (Siglec-10) receptor. We aimed to identify the features of CD52 glycan that underlie its bioactivity. Analysis of native CD52 purified from human spleen revealed extensive heterogeneity in *N-*glycosylation and multi-antennary sialylated *N-*glycans with abundant polyLacNAc extensions, together with mainly di-sialylated *O-*glycosylation type structures. Glycomic (porous graphitized carbon-ESI-MS/MS) and glycopeptide (C8-LC-ESI-MS) analysis of recombinant soluble human CD52-immunoglobulin Fc fusion proteins revealed that CD52 bioactivity was correlated with a high abundance of tetra-antennary α-2,3/6 sialylated *N-*glycans. Removal of α-2,3 sialylation abolished bioactivity, which was restored by re-sialylation with α-2,3 sialyltransferases. When glycoforms of CD52-Fc were fractionated by anion exchange MonoQ-GL chromatography, bioactive fractions displayed mainly tetra-antennary, α-2,3 sialylated *N-*glycan structures and a lower relative abundance of bisecting GlcNAc structures compared to non-bioactive fractions. In addition, *O-*glycan core type-2 di-sialylated structures at Ser12 were more abundant in bioactive CD52 fractions. Understanding the structural features of CD52 glycan required for its bioactivity will aid its development as an immunotherapeutic agent.

## Introduction

CD52 is a glycoprotein composed of only 12 amino acid extensively modified by both N-linked and possible *O*-linked glycosylation, anchored by glycosylphosphatidylinositol (GPI) to the surface of leukocytic, and male reproductive cells (1, 2). The conserved CD52 peptide backbone probably functions only as a scaffold for presentation of the large N-linked glycan, which masks the small GPI-anchored peptide and acts as the prime feature of the CD52 antigen with respect to cell-cell contacts (1, 2). This notion is supported by the recent discovery of the immune suppressive role of soluble CD52 *in vitro* and *in vivo* (3–5).

Activated human T cells with high expression of CD52 were found to exhibit immune suppressive activity via phospholipase C-mediated release of soluble CD52, which was shown to bind to the inhibitory sialic acid-binding immunoglobulin (Ig)-like lectin-10 (Siglec-10) receptor on neighboring T cell populations (3). This sialic acid interaction was subsequently shown to require initial binding of soluble CD52 glycan to the damage-associated molecular pattern (DAMP) protein, high-mobility group box 1 (HMGB1). Complexing of soluble CD52 with HMGB1 promoted binding of the CD52 N-glycan, preferentially in α-2,3 sialic acid linkage, to Siglec-10 (4).

In the only previous mass spectrometric analysis, the *N-*glycans on human leukocyte CD52 exhibited extensive heterogeneity with multi-antennary complexes containing core α-1,6 fucosylation, abundant polyLacNAc extensions, and variable sialylation (6). With recent insights into the function of soluble CD52, and its potential as an immunotherapeutic agent, the glycan structure-function determinants of CD52 warrant more detailed investigation. In particular, although the CD52 *N-*glycan is known to be required for bioactivity (3, 4), its structure is not fully elucidated and the glycoforms required for bioactivity have not been identified. In addition, even with a total of six potential serine or threonine attachment sites, *O-*glycosylation of CD52 has not been analyzed. We aimed therefore to identify the structural features of CD52 glycan required for its bioactivity using both purified native human CD52 and recombinant soluble CD52 expressed as a fusion protein with immunoglobulin Fc.

## Materials and Methods

### Human Blood and Spleen Donors

Cells were isolated from human blood buffy coats (Australian Red Cross Blood Service, Melbourne, VIC, Australia) or blood of de-identified healthy volunteers with informed consent through the Volunteer Blood Donor Registry of The Walter and Eliza Hall Institute of Medical Research (WEHI), following approval by WEHI and Melbourne Health Human Ethics Committees. Peripheral blood mononuclear cells (PBMCs) were isolated from fresh human blood on Ficoll/Hypaque (Amersham Pharmacia, Uppsala, Sweden), washed in phosphate-buffered saline (PBS) and re-suspended in Iscove's Modified Dulbecco's medium (IMDM) containing 5% pooled, heat-inactivated human serum (PHS; Australian Red Cross, Melbourne, Australia), 100 mM non-essential amino acids, 2 mM glutamine, and 50 μM 2-mercaptoethanol (IP5 medium).

A cadaveric spleen was obtained via the Australian Islet Transplant Consortium and experienced coordinators of Donate Life from a heart-beating, brain dead previously healthy donor, with informed written consent of next of kin. All studies were approved by WEHI Human Research Ethics Committee (Project 05/12).

### Purification of Native CD52 From Human Spleen

Frozen human spleen tissue (10 mg) was homogenized with three volumes of water as per described in Xia et al. (1). In brief, homogenate was mixed with methanol and chloroform 11:5.4 volumes, respectively. Samples were left to stir for 30 min and allowed to stand for 1 h. The upper (aqueous) phase was collected, evaporated, dialyzed, and freeze dried. NHS-activated Sepharose 4 Fast Flow resin was incubated with 1 mg of purified anti-CD52 antibody in 0.5 mL of PBS for 3 h at RT. The mixture was incubated overnight at 4°C and quenched with 1 M ethanolamine. A Bio-Rad 10-mL Poly-Prep column was used for packing and resins were washed with sequential treatment of 5 mL of PBS, 5 mL of pH 11.5 diethylamine, and 5 mL of PBS/0.02% sodium azide. The column was stored at 4°C in 5 mL of PBS/0.02% sodium azide before use. Spleen extracts were solubilized with 2 mL of 2% sodium deoxycholate in PBS, and then added to the packed column and washed with 5 mL of PBS containing 0.5% sodium deoxycholate. The sample was eluted with six times 500 μl of elution buffer (50 mM diethylamine, 500 mM NaCl, pH 11.5) containing 0.5 % sodium deoxycholate. The eluate was collected, neutralized with 50 μl of HCl (0.1 M) and dialyzed against PBS and water.

### CD52 Recombinant Proteins

Human CD52-Fc recombinant proteins; CD52-Fc I (Expi293), CD5-Fc II (FreeStyle HEK293F), and CD52-Fc III (Expi293) were produced as described (3). The signal peptide sequences joined to human IgG1 Fc were constructed with polymerase chain reaction (PCR) then digested and ligated into a FTGW lentivirus vector or pCAGGS vector for the transfection of HEK293F and Expi293 cells. The construct included a flexible GGSGG linker, a strep-tag II sequence for purification (7), and a cleavage sites for Factor Xa protease between the signal peptide and Fc molecule. The recombinant proteins were purified from the medium by affinity chromatography on Streptactin resin and eluted with 2.5 mM desthiobiotin (3).

### ^3^H-Thymidine Incorporation Assay

PBMCs are primary cells and cannot be cultured for more than one passage under normal conditions. PBMCs (2 × 10^5^ cells/well) in IP5 medium were incubated for up to 3 d at 37°C in 5% CO_2_ in 96-well round-bottomed plates with or without the activating antigen, tetanus toxoid (10 Lyons flocculating units per ml), and various concentrations of CD52-Fc or control Fc protein, in a total volume of 200 μL. To measure cell proliferation, the radioactive nucleoside, ^3^H-thymidine (1 μCi), was added for the last 16 h of incubation. ^3^H-thymidine is incorporated into newly-synthesized DNA during mitotic cell division. The cells were collected and radioactivity in DNA measured by scintillation counting.

### ELISpot Assay

An IFN-γ ELISpot assay was employed as a further means to demonstrate the immune suppressive activity of CD52-Fc. PBMCs (2 × 10^5^ cells/well) were cultured in 200 μL of IP5 medium in triplicate wells of a 96-well ELISpot plate (PVDF MultiScreen) from Merck Millipore (Bayswater, Australia) containing anti-IFN-γ monoclonal antibody pre-bound (1 μg/mL) at 4°C. Tetanus toxoid (10 Lfu/mL) was added to the wells together with CD52-Fc I, CD5-Fc II or CD52-Fc III (5, 25, and 50 μg/mL). After 24 h, cells were removed by washing and IFN-γ spots, denoting single T cells, were developed by incubation with biotinylated anti-IFN-γ antibody (1 μg/mL) followed by streptavidin-alkaline phosphatase and BCIP/NBT color reagent (Resolving Images, Melbourne, Australia).

### Lectin ELISA

We have previously (4) used *Maackia amurensis* and *Sambucus nigra* lectins to distinguish CD52-Fc glycans containing, respectively, sialic acid in α-2,3 and α-2,6 linkage with galactose (8, 9). Here we used *Maackia amurensis* (MAA-I/MAL-I; Vector Laboratories, Burlingame, USA) to identify the α-2,3 linkage. A 96-well flat-bottom plate was coated with 20 μg/mL of MAL-1 overnight at 4°C and subsequently blocked with 200 μl of 1 % BSA for 1 h. After washing with PBS, CD52-Fc I, CD52-Fc II, or CD52-Fc III (20 μg/mL) were added and incubated at RT for 1 h and washed twice with PBS. After washing with PBS, 50 μl of a 1:1,000 dilution of HRP-conjugated antibody to CD52 (Campath H1; 1 μg/mL) was added and incubated at RT for 1 h. 50 μl of 3,3′5,5′-tetramethylbenzidine (TMB) substrate was added and color development stopped by addition of 50 μl of 0.5 M H_2_SO_4_. Absorbance was measured at 450 nm in a Multiskan Ascent 354 microplate photometer (Thermo Labsystems, San Francisco, USA).

### De-sialylation and Re-sialylation of Recombinant CD52-Fc Protein

De-sialylation and re-sialylation of recombinant CD52-Fc III proteins were performed by a modification of the method of Paulson and Rogers (10). Briefly, CD52-Fc (500 μg/each) was incubated with *Clostridium perfringens* type V sialidase (50 mU/mL) for 3 h at 37°C to remove all types of sialic acids. Samples were then passed through a Protein G-Sepharose column, which was washed twice with PBS before the bound protein was eluted with 0.1 M glycine-HCl, pH 2.8 into 1 M Tris-HCl, pH 8.0, followed by dialysis against PBS. Binding to MAL-I lectin was performed to confirm removal of sialic acids. CD52-Fc III from Expi293 cells was then incubated with either of two sialyltransferases, PdST6GalI which restores sialic acid residues in α-2,6 linkage with underlying galactose or CstII which restores sialic acid residues in α-2,3 linkage with galactose, in the presence of 0.46 mM-0.90 mM CMP-N-acetylneuraminic acid sodium salt (Carbosynth, Compton Berkshire, United Kingdom) for 3 h at 37°C. The different CD52-Fc (III) proteins with different linkages (α-2,3 or α-2,6) were passed through Protein G-Sepharose columns, washed twice with PBS and eluted with 0.1 M glycine-HCl, pH 2.8, into 1 M Tris-HCl, pH 8.0, followed by dialysis against PBS. Samples were freeze-dried, re-suspended in PBS at 200 μg/mL and stored at −20°C.

### Fc Fragment Removal

CD52-Fc III recombinant protein fractions (50–200 μg) were incubated with 4 μL of Factor Xa protease (purified from bovine plasma, New England Biolabs, Ipswich, USA) in a total volume of 1 mL of cleavage buffer (20 mM Tris-Hcl, pH 8, 100 mM NaCl, 2 mM CaCl_2_). Samples were incubated overnight at RT. Samples were mixed three times with Protein G-Sepharose beads for 1 h at RT and centrifuged at 10,000 rpm for 15 min. Fc fragment removal was confirmed by Western blot using anti-human IgG (Fc specific produced in goat; Sigma Aldrich, St. Louis, USA) and anti-CD52 (rabbit) antibodies (Santa Cruz Biotechnology, Dallas, USA).

### *N*- and *O*- Linked Glycan Release for Mass Spectrometry Analysis

Mono Q fractionated and whole (non-fractionated) recombinant CD52-Fc III were dot-blotted on a PVDF membrane. Soluble CD52 with the Fc removed was kept in-solution prior to *N-*glycan release by an overnight incubation with 2.5 units of N-glycosidase F (PNGase F from *Elizabethkingia miricola*, Roche, Basel Switzerland) at 37°C followed by a NaBH4 reduction (1 M NaBH4, 50 mM KOH) for 3 h at 50°C. The *O-*glycans were subsequently released by overnight reductive β-elimination using 0.5 M NaBH4, 50 mM KOH at 50°C. The released and reduced *N*- and *O*-glycans were thoroughly desalted prior to the LC-MS/MS as described previously (11).

### Mass Spectrometry and Data Analysis of Released Glycans

The separation of glycans was performed by using a porous graphitized carbon (PGC) column (5 μm particle size, 180 μm internal diameter × 10 cm column length; Hypercarb KAPPA Capillary Column (Thermo Scientific, Waltham, USA), operated at a constants flow rate of 4 μl/min using a Dionex Ultimate 3000 LC (Thermo Scientific). The separated glycans were detected online using liquid chromatography-electrospray ionization tandem mass spectrometry (LC-ESI-MS/MS) using an LTQ Velos Pro mass spectrometer (Thermo Scientific). The PGC column was equilibrated with 10 mM ammonium bicarbonate (Sigma Aldrich) and samples were separated on a 0–70% (v/v) acetonitrile in 10 mM ammonium bicarbonate gradient over 75 min. The ESI capillary voltage was set at 3.2 kV. The full auto gain control was set to 80,000 kV. MS1 full scans were made between *m/z* 600–2,000. All glycan mass spectra were acquired in negative ion mode. The LTQ mass spectrometer was calibrated with a tune mix (Pierce^TM^ ESI negative ions, Thermo Scientific) for mass accuracy of 0.2 Da. The CID-MS/MS was carried out on the five most abundant precursor ions in each full scan by using 35 normalized collision energy. Possible monosaccharide compositions were provided by GlycoMod (Expasy, http://web.expasy.org/glycomod/) based on the molecular mass of glycan precursor ions (12). Analysis of MS/MS spectra was performed with Thermo Xcalibur Qual browser software. Possible glycan structures were identified based on diagnostic fragment ions 368 for core fucosylation and others as reported (13), and B/Y- and C/Z-glycan fragments in the CID-MS/MS spectra. A mass tolerance of 0.2 Da was allowed for both the precursor and product ions. The relative abundances of the identified glycans were determined as a percentage of the total peak area from the MS signal strength using area under the curve (AUC) of extracted ion chromatograms of glycan precursor ion (14).

### Profiling the *N*- and *O*- Glycans on the CD52 Peptide

MonoQ fractionated and unfractionated CD52 glycoforms without the Fc were desalted on C18 micro-SPE stage tips (Merck-Millipore, Burlington, USA). Elution was performed with 90% acetonitrile (ACN) and samples were dried and redissolved in 0.1% Formic acid (FA). The desalted CD52 glycopeptides were analyzed by ESI-LC-MS in positive ion polarity mode using a Quadrupole-Time-of-flight (Q-TOF) 6538 mass spectrometer (Agilent technologies, Mulgrave, Australia)-HPLC (Agilent 1260 infinity). In parallel experiments, *N-*glycosidase F was used to remove *N-*glycans from some samples of CD52 (with a resulting Asn->Asp conversion i.e., +1 Da) to enable better ionization of the highly heterogeneous and anionic CD52 glycopeptides. The *N-* and *O-*glycan occupancy was (500 ng) were injected onto a C8 column (ProteCol C8, 3 μm particle size, 300 A pore size, 300 nm inner diameter 10 cm length; SGE analytical science). The HPLC gradient was made starting with 0.1% FA with a linear rise to 60% (v/v) ACN 0.1% FA over 30 min. The column was then washed with 99% ACN (v/v) for 10 min before re-equilibration with 0.1% FA for another 10 min. The flow rate was set to 4 μL/min with an optimized fragmentor positive potential of 200 V with the following MS setting: m/z range 400–2,500, nitrogen drying gas flow rate 8 L/min at 300°C, nebulizer pressure was 10 psi, capillary positive potential was 4.3 kV, skimmer potential was 65 V. The mass spectrometer was calibrated with a tune mix (Agilent technologies) to reach a mass accuracy typically better than 0.2 ppm. MassHunter workstation vB.06 (Agilent technologies) was used for analysis and deconvolution of the resulting spectra. The previously determined glycans from the PGC-ESI-MS/MS analysis were used to guide the assignment of glycoforms to deconvoluted CD52 peptides based on the accurate molecular mass.

### Mono Q Column Fractionation

CD52-Fc III was diluted into 5 mL 50 mM Tris-HCl, pH 8.3, and applied to a Mono Q column (Mono Q 5/50 GL, GE Lifesciences, Parramatta, Australia). The column was washed with 10 column volumes of 50 mM Tris-HCl, pH 8.3, and then eluted with 50 column volumes of 50 mM Tris-HCl, 500 mM NaCl, pH 8.3 in 0.5 mL fractions. Fractions were then collected and analyzed by isoelectric focusing (IEF).

### IEF

Novex pH 3–10 IEF gels (Life Technologies, Carlsbad, USA) were used for pI determination. CD52-Fc fractions were loaded with sample buffer and run at 100 V for 2 h, then at 250 V for 1 h and, finally, the voltage was increased to 500 V for 30 min. After electrophoresis, the gel was carefully transferred to a clean container, washed and fixed with 20% trichloroacetic acid (TCA) for 1 h at RT, rinsed with distilled water, stained with colloidal Coomasie blue (Sigma Aldrich) for 2 h at RT, and thoroughly de-stained with distilled water.

### Sequential Sialidase Treatment

*N-*glycans released from cleaved CD52 (2 μg) were treated with α-2-3-specific sialidase (1 mU, Sigma Aldrich) and broad (α-2-3,6,8 sialidase-reactive) sialidase *V. cholera* (1 mU, Sigma Aldrich). Both reactions were carried out in 50 mM sodium phosphate reaction buffer at 37°C for 3 h. De-sialylated CD52 *N-*glycans were dried and solubilised in water for downstream MS analysis. Fetuin was used as positive control for successful sialic acid removal since, like cleaved CD52, this model glycoprotein carries multi-antennary sialylated *N-*glycans.

### EThcD Fragmentation for *O*-Glycan Site Localization on the CD52 Peptide

Fractionated CD52 glycoforms were treated with PNGase F prior to *O-*glycan site localization analysis. CD52 peptides were analyzed using a Dionex 3500RS nanoUHPLC coupled to an Orbitrap Fusion™ Tribrid™ Mass Spectrometer in positive mode with the same LC gradient mentioned in “Profiling the *N-* and *O-* glycans on intact CD52,” but with a nano-flow (250 nL/min). The following MS settings were used: spray voltage 2.3 kV, 120 k orbitrap resolution, scan range *m/z* 550–1,500, AGC target 400,000 with one microscan. The HCD-MS/MS used 40% nCE. Precursors that resulted in fragment spectra containing diagnostic oxonium ions for glycopeptides i.e., *m/z* 204.08671, 138.05451, and 366.13961, were selected for a second EThcD (nCE 15%) fragmentation. The analysis of all fragment spectra was carried out using Thermo Xcalibur Qual browser software with the aid of Byonic (v2.16.11, Protein Metrics Inc, Cupertino, USA) using the following parameters: precursor mass tolerance 6 ppm, fragment mass tolerance 1 Da and 10 ppm to respectively, account for possible proton transfer during ETD fragment formation and the MS/MS resolution, deamidated (variable), and two core type 2 *O-*glycans, previously seen in intact mass analysis.

Data are expressed as mean ± standard deviation (SD). The significance of differences between groups was determined by ANOVA, *post-hoc* comparisons of pairs and Bonferroni correction, with Prism software (GraphPad Software). *p* < 0.05 was used throughout as the significance threshold.

## Results

### Human Spleen-Derived CD52 Exhibits Extensive *N*- and *O*-Glycosylation Heterogeneity

**Figure 1:**
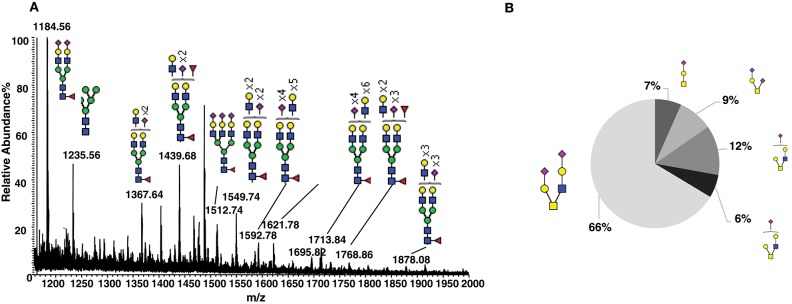
Glycosylation analysis of human spleen CD52. **(A)** Summed MS profile of released *N-*glycans from CD52 purified from human spleen tissue. **(B)** Distribution of *O*-linked glycans released from human spleen CD52. CD52 was purified from one healthy donor spleen.

To characterize the natural glycosylation of human CD52, we purified CD52 from human spleen and performed a comprehensive analysis of released *N-* and *O-*glycans by porous-graphitized carbon (PGC)-ESI-MS/MS (Figures 1A,B). We confirmed high *N-*glycosylation heterogeneity, expressed as multi-antennary sialylated *N-*glycans with abundant polyLacNAc extensions (Figure 1A). Similar *N*- glycans have been previously reported for natural occurring human CD52 (5). The *O-*glycosylation profile was characterized as core type 1 and core type 2 sialylated structures with mainly (66%) di-sialylated core type 2 *O-*glycans (Figure 1B). This glycan heterogeneity raises the question whether particular bioactive glycoforms of CD52 exist and whether such heterogeneity is reflected in the recombinant form of human CD52.

**Figure 2:**
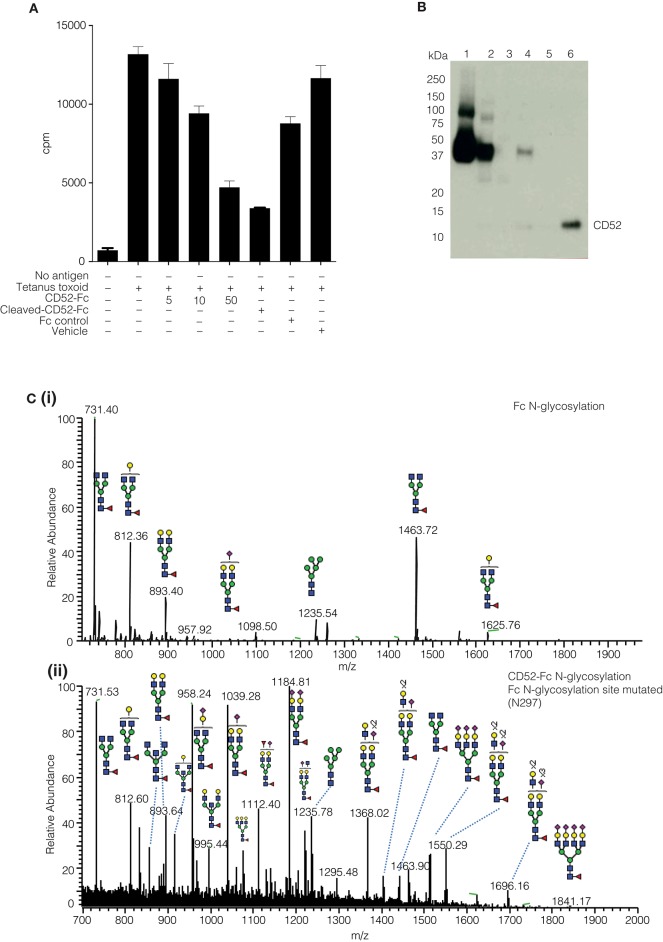
Comparative N-glycoprofiling of recombinant human IgG Fc and CD52. **(A)** Proliferation of human PBMCs (^3^H thymidine uptake) followed 5 days incubation with tetanus toxoid (10 LfU), histograms show mean ± SD of within-assay triplicates, in the presence of different concentration of proteins (CD52-Fc 5, 10, 50 μg/ml; Cleaved CD52-Fc 50 μg/ml and Fc control 50 μg/ml). The Fc component was cleaved from CD52-Fc with Factor Xa. **(B)** Factor Xa treated-CD52 was analyzed by Western blotting with anti-CD52-HRP antibody (Campath-H1). **(C)** Summed MS profile of N-glycans released from the Fc (I) and CD52 (II); the latter variant was generated by introducing a point mutation (A297N) into the conventional Fc N-glycosylation site.

The yield of purified native soluble CD52 was insufficient to enable us to pinpoint the bioactive glycoforms on the naturally occurring glycoprotein. Therefore, we engineered human CD52 as a recombinant fusion protein conjugated with an IgG1 Fc fragment as described (3). Previously, we demonstrated the ability of recombinant CD52-Fc, but not its Fc component, to suppress a range of immune functions (3, 4). The two recombinant human CD52-Fc batches we generated for this study recapitulated the previously observed immuno-suppressive bioactivity (Figure 2A). However, the Fc has a single *N-*linked glycosylated site at N297 (Figure 2Ci), which had to be considered in characterizing and assessing the impact of the *N*-glycosylation of recombinant CD52-Fc. This was addressed in two ways: (i) by analyzing a recombinant form of human CD52-Fc in which Fc contained a N297A mutation, allowing analysis of CD52 *N*-glycosylation profile at the released glycan level without interference from the Fc *N-*glycan (Figure 2Cii), and (ii) by removal of the Fc component from CD52-Fc by Factor Xa proteolysis of a cleavage site appropriately incorporated in the CD52-Fc construct, as shown by a Western blot using a specific antibody for CD52 (Figure 2B).

### Bioactive Recombinant CD52 Glycoforms Displays More Abundant tri- and Tetra-Antennary Sialylated *N*-Glycans

**Figure 3:**
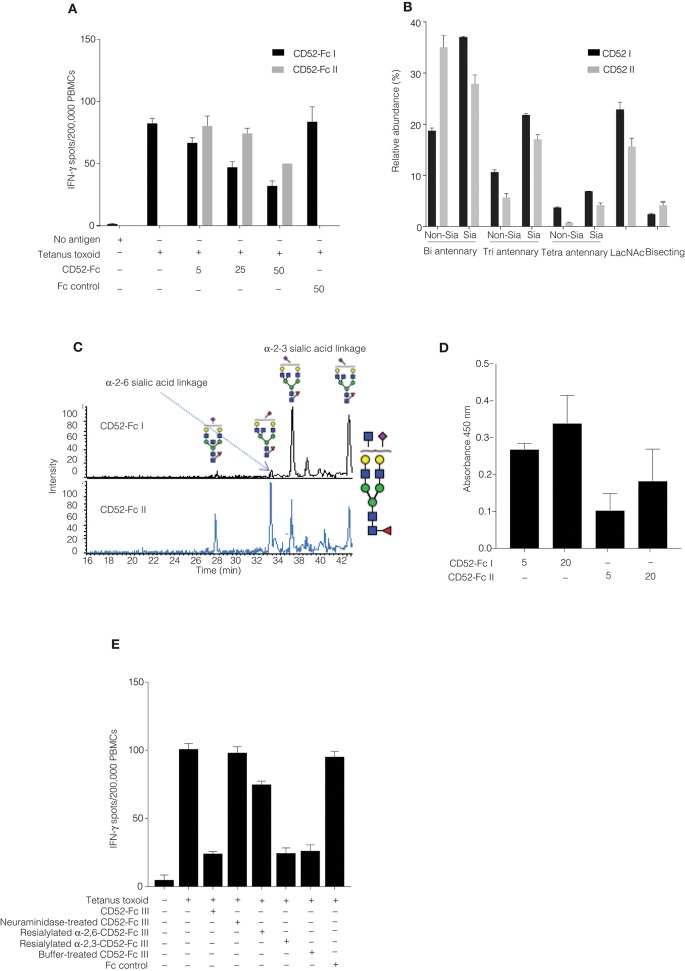
Comparison of recombinant human CD52-Fc variants (I, II, and III) with different immunosuppressive activities. **(A)** IFN–γ production measured by ELISpot assay from human PBMCs (2 × 10^6^) in 200 μL/well. Samples were incubated with no antigen or tetanus toxoid in the presence of two different preparations of CD52-Fc (CD52 I or CD52 II; 5, 25, and 50 μg/ml). **(B)** *N*-linked glycans released from cleaved CD52 I and CD52 II. The abundance of each *N-*glycan class is the sum of all EICs measured for all glycans in that class relative to the total of all EICs observed for all *N-*glycans. **(C)** EIC of *m/z* 1140.4^2−^ (GlcNAc_5_Man_3_Gal_2_NeuAc_1_) demonstrating the PGC-based separation of sialo-glycan isomers observed in CD52 I and CD52 II. **(D)** Binding of CD52-Fc I and CD52-Fc II (5 and 20 μg/ml) to the α-2,3 sialic acid recognizing lectin MAL-1. **(E)** ELISpot assay showing activity of CD52-Fc III reconstituted with sialic acid in α2-6, α2-3, and α2-8 linkages with galactose. The data points in **(A,D,E)** are plotted as mean ± SEM of three independent replicate experiments. Data in B and C are mean ± SDs (*n* = 3). ANOVA, *post-hoc* comparisons of pairs and Bonferroni correction were used to test for significant difference between group means.

We had noted that the specific bioactivity of recombinant CD52-Fc varied from batch to batch. Therefore, we compared two CD52-Fc variants made in different host cells, here referred to respectively, as CD52-Fc I (from Expi 293 cells) and CD52-Fc II (from HEK 293F cells), which displayed higher and lower immunosuppressive activity (Figure 3A).

*N*-glycans were released *via* in-solution treatment with PNGase F and subsequently analyzed by PGC-ESI-MS/MS (9). *N*-glycans on cleaved CD52 I had greater relative abundances of bi-, tri- and tetra- antennary sialylated glycans compared to CD52 II (Figure 3B). Also, CD52 I displayed a significantly higher relative abundance of sialylated structures possibly containing LacNAc moieties (Figure 3B). Not only the numbers of antennae, but also their degree of sialylation differed between the two recombinant CD52 glycoforms: tetra-sialylated *N*-glycans were significantly more abundant in CD52 I (6.9 ± 0.1%) compared to CD52 II (4.2 ± 0.6; *p* < 0.05). In contrast, CD52 II displayed significantly greater abundance of non-sialylated bi-antennary and bisecting structures (35 and 4% compared to 19 and 2%, respectively; Figure 3B).

After the removal of Fc, recombinant CD52 I and CD52 II were then subjected to high-resolution intact peptide analysis using C8-LC-ESI-MS. Both proteins showed *N*-glycosylation profiles similar to those of released glycans. The high resolution of the Q-TOF instrumentation used even in the high m/z range enabled the identification of very elongated sialylated antennary structures including searching for *N*-glycans carrying Lewis-type structures (antenna-type fucosylation). The experimental isotopic distribution of both variants of recombinant CD52 matched the theoretical isotopic distribution of the 90% tri-sialylated (non-Lewis fucosylated) CD52 glycoforms, indicating that the main glycoforms of recombinant CD52 do not carry Lewis-type fucosylation (Supplementary Figure 1A). The more bioactive CD52 I displayed a higher level of multi-antennary sialylated and possible LacNAc elongated structures (Supplementary Figure 1B).

### α-2,3 Sialylated *N*-Glycans Are Indispensable for CD52 Activity

CD52 *N-*glycans displaying α-2,3 sialylation preferentially bind to Siglec-10 (4). PGC-ESI-MS/MS glycan analysis and MAL-I lectin blotting were used to identify any differences in sialic acid linkage between the two variants of recombinant CD52-Fc (CD52-Fc I and CD52-Fc II). MAL-I preferentially recognizes α-2,3 sialic acid linked tri- and tetra-sialylated *N*-glycans (15). Despite the high separation power of PGC for sialoglycans, this technique has difficulty resolving very large multi-antennary sialylated glycans, but can easily discriminate between α-2,3 and α-2,6-sialylation on the more common bi- and tri-antennary *N*-glycans. Several abundant bi-antennary α-2,3 sialoglycans were observed on CD52 I. For one sialylated glycan, *m/z* 1140.4^2−^ (GlcNAc_5_Man_3_Gal_2_NeuAc_1_), only the α-2,3 sialic acid glycan isomer was observed on CD52 I. On the other hand, the less bioactive CD52 II carried both α-2,3 and α-2,6 sialo-*N*-glycans (Figure 3C). This differential sialyl linkage presentation between the two recombinant CD52 variants was supported by MAL-I lectin binding, which was higher for the more bioactive CD52-Fc I (Figure 3D). The importance of α-2,3 sialylation for bioactivity of CD52-Fc was confirmed in a parallel experiment in which the immunosuppressive activity of sialidase-treated and re-sialylated CD52-Fc was determined relative to the original recombinant variant. Treatment of CD52-Fc with sialidase completely abolished its immunosuppressive activity, which was fully restored upon re-sialylation with α-2,3, but not α-2,6 (Figure 3E). Overall, these findings indicate that the bioactivity of CD52-Fc is associated with the presence of α-2,3-linked tetra-sialylated *N-*glycans found on CD52.

### Active CD52 Glycoforms Resolved by Anion Exchange Chromatography

**Figure 4:**
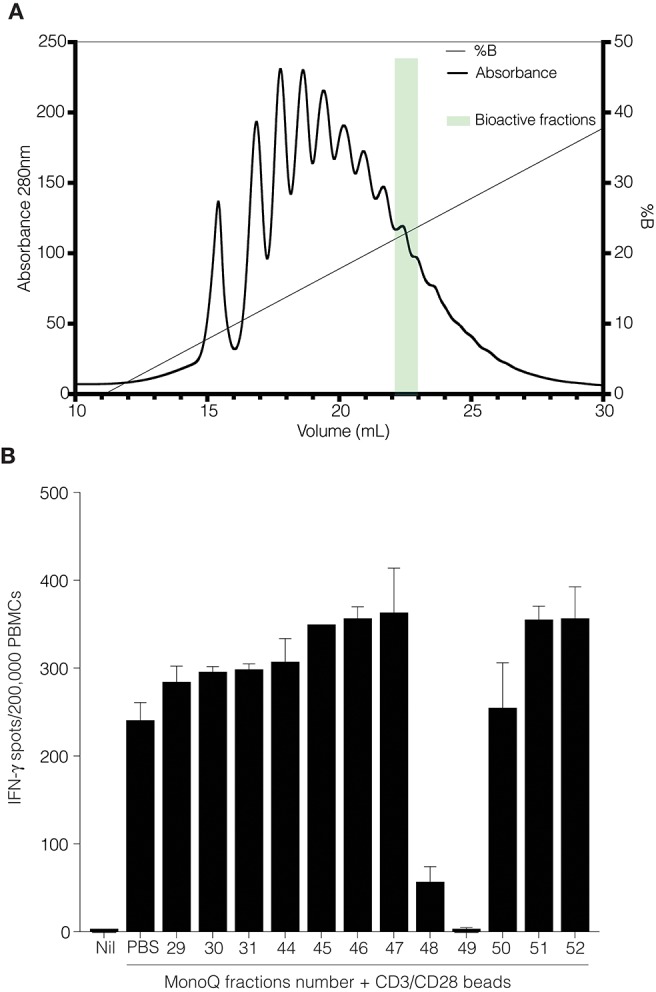
CD52-Fc after fractionation by anion-exchange chromatography. **(A)** Anion exchange chromatography on a MonoQ-GL column fractionated the recombinant human CD52-Fc III into a gradient of anionic glycoforms displaying a spectrum of pI (see Supplementary Figure 2). **(B)** IFN–γ ELISpot assay with 2 × 10^6^ PBMCs in 200 μL/well incubated with no antigen or with anti-CD3/CD28 antibody Dynabeads in the presence of recombinant human CD52-Fc fractions (F29–F52; 5 μg/ml).

We performed anion exchange chromatography on a MonoQ column in order to separate recombinant CD52-Fc III variants based on their degree of sialylation, with the aim of identifying the most bioactive forms (Figure 4A). The increasing degree of sialylation [decreasing isoelectric point [pI]] of CD52-Fc in the collected fractions was confirmed by isoelectric focusing (IEF) (Supplementary Figure 2) and mass spectrometry. The released *N-*glycans from fractions 46 to 51 (F46-F51) exhibited a gradual increase in sialic acid content, and structures containing a higher number of antennae (Table 1), as shown also from intact glycopeptide analysis (Supplementary Figure 3). Released and intact glycan analysis from fraction 30 revealed various GlcNAc and Gal capped structures and a complete absence of sialic acid moieties (Table 1 and Supplementary Figure 3). Remarkably, only two fractions, F48 and F49, with pIs in the 5–6 range, displayed significant immunosuppressive activity (Figure 4B). The adjacent fractions were not bioactive, even at higher concentrations of protein (Supplementary Figures 4A,B). These late-eluting, uniquely bioactive fractions (F48–49) were highly enriched (60–70%) in tri- and tetra-sialylated glycans.

**Table 1:**
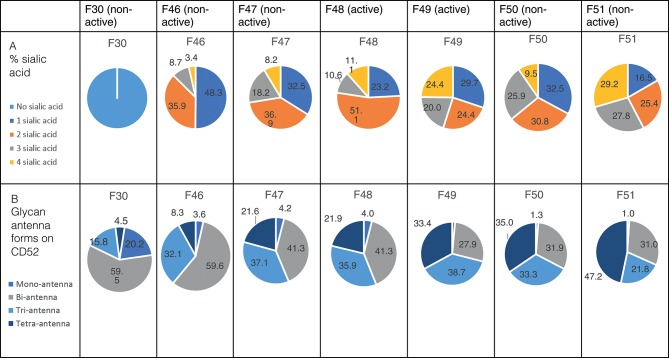
Sialic acid content and antennae distribution of recombinant human CD52 fractions separated by anion chromatography.

***(A)** (upper panel) The total number of sialic acid residues and **(B)** (lower panel) The antennae distribution identified on CD52 fractions (F30, F46, F47, F49, F50, and F51) using PGC-ESI-MS/MS*.

### Active CD52 MonoQ Fractions Are Enriched With α-2,3 Sialylated Structures

**Figure 5:**
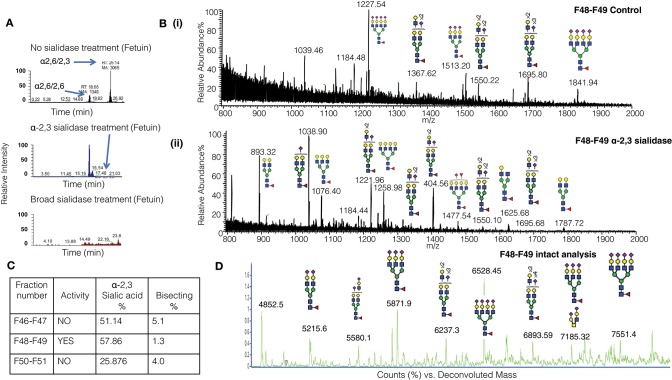
Sialic linkage analysis of active monoQ active fractions. **(A)** EICs of the di-sialylated *N*-glycan *m/z* 1111.4^2−^ after sequential α-2,3 sialidase treatment of bovine fetuin, known to carry tri-antennary α-2,3-sialylated *N*-glycans. The EICs assess the removal of each of the sialic acid residues. **(B)** Summed MS of all *N*-glycans observed for the active CD52 fractions F48 and F49 before (i) and after treatment with α-2,3-specific sialidase (ii). **(C)** Summary of the degree of α-2,3 sialylation and bisecting GlcNAcylation of late-eluting MonoQ fractions of particular interest. **(D)** High-resolution intact mass analysis of the immune suppressive CD52 fractions (F48/F49).

It is challenging to determine the sialylation linkages of large, multi-sialylated *N-*glycans by mass spectrometry. Therefore, differences in sialic acid linkage of active and adjacent non-active MonoQ fractions were probed by α-2,3-specific sialidase treatment. The linkage-specific activity of α-2,3 sialidase was confirmed on bovine fetuin as a control protein; specific removal of α-2,3-linked sialic acid residues from this known bi-antennary sialylated glycan *m/z* 1111.5^2−^ was demonstrated (Figure 5A). The glycan products resulting from α-2,3 sialidase treatment of the active fractions of CD52 were determined via PGC-ESI-MS/MS (Figures 5Bi,ii). The active MonoQ fractions (F48/F49) had a higher proportion of α-2,3 sialic acid (58%) compared to adjacent earlier (F46, F47) and later (F50, F51) eluting fractions (51 and 25%, respectively) and less bisecting structures than the adjacent non-active fractions (1%, compared to 4 and 5%, respectively; Figure 5C). Finally, the profile of the most active CD52 fractions at the intact peptide level supported a predominance of tri- and tetra-antennary sialylated structures (Figure 5D).

### The Highly Anionic MonoQ Fractions Are Enriched in *O*-Sialylated Glycans

**Figure 6:**
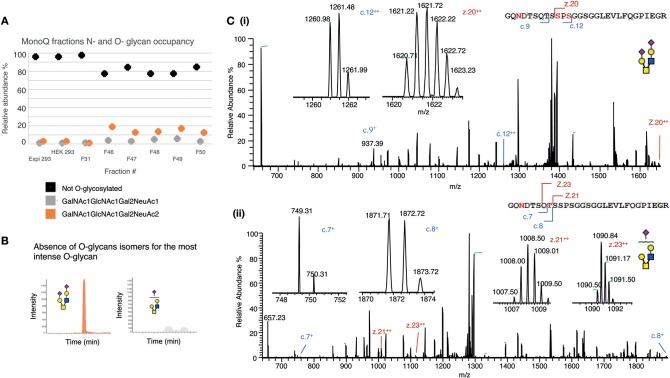
Mapping the *O*-glycosylation of recombinant human CD52. **(A)** *N-* and *O-*glycan occupancy of CD52 I, CD52 II, and selected MonoQ fractions (F31 and F46–F51) measured at the protein level after de-*N-*glycosylation. **(B)** PGC resolution of *O-*glycosylated isomers from active fractions *m/z* 665.2^2−^ (GalNAc_1_GlcNAc_1_Gal_2_NeuAc_2_) and *m/z* 1040.4^1−^ (GalNAc_1_GlcNAc_1_Gal_2_NeuAc_1_). **(C)** EThcD-MS/MS based site localization analysis showing the peptide backbone fragments and the ions diagnostic of the amino acid site for both aforementioned *O-*glycans. Fragmentation to c and z ions are shown that indicate that (i) di-sialylated *O*-glycans were conjugated to Ser12, and possibly Ser10, whereas (ii) the mono-sialylated *O*-glycans are found on Thr8.

Initially, *O*-glycosylation analysis of de-*N*-glycosylated CD52 at the intact peptide level revealed that both variants of recombinant CD52 (CD52 I and CD52 II) had very low (4%) *O*-glycan occupancy (Figure 6A), casting doubt on the relevance of *O-*glycosylation for CD52 activity. Non-deamidated signatures were absent in the spectra for both CD52 I and II, indicating that the CD52 peptides were fully *N*-glycosylated (Figure 6A, black symbols). Like human spleen CD52, the recombinant CD52 proteins were found to contain mainly core type 2 *O*-glycans with one or two sialic acid residues (Figure 6A, gray and orange symbols, respectively). Sialylated core type 1 *O-*glycans were also identified albeit at very low abundance (<0.5%) (data not shown). Interestingly, the most anionic MonoQ CD52 fractions (F46-F51) had a considerably higher *O*-glycan occupancy (15–20%) compared to the original non-fractionated CD52 (4%). Extracted ion chromatograms (EIC) of the bioactive fractions (F48 and F49) showed an absence of sialo-isomers for the most abundant *O*-glycan structure *m/z* 665.2^2−^ (GalNAc_1_GlcNAc_1_Gal_2_NeuAc_2_), but not for m/z 1040.4^1−^(GalNAc_1_GlcNAc_1_Gal_2_NeuAc) (Figure 6B). Finally, *O*-glycan site localization was determined by electron transfer/higher-energy collision dissociation (EThcD), which provided c and z ions, allowing the conclusion that di-sialylated *O*-glycans were conjugated to Ser12, and possibly Ser10, whereas the mono-sialylated *O*-glycans were only found on Thr8 (Figures 6Ci,ii).

## Discussion

In this study, we determined that CD52 from human spleen and recombinant forms of human CD52-Fc carry *N-*glycans that display complex type core fucosylation, abundant sialylation, and LacNAc extensions. These features corroborate a previous report (6) on the *N-*glycan of human spleen CD52, but we extended this in several ways. By comparing two recombinant CD52-Fc glycoproteins that differed in specific bioactivity, made in different host cells, we found that the more bioactive form had a significantly higher abundance of tetra-sialylated *N-*glycan structures with α-2,3 sialic acid linkage. The less bioactive form, on the other hand, exhibited significantly higher bisecting GlcNAc structures. By MonoQ anion exchange chromatography, CD52-Fc was separated into a gradient of anionic glycoforms, which exhibited distinctly different immunosuppressive activities. Again, the most bioactive glycoforms uniquely displayed an abundance of tri- and tetra-sialylated glycans (60–70%), high levels of α-2,3 sialylation (58%), and an absence of bisecting GlcNAcylation. Moreover, the most anionic tri- and tetra-sialylated *N-*glycopeptides had a unique abundance in core type 2 di-sialylated *O-*glycan on Ser 12.

Both glycan- and glycopeptide-based analytical approaches were used to correlate CD52 glycan structure with CD52 bioactivity. The glycan approach depended on the high resolving power of PGC columns to separate glycan isomers and isobaric structures. It was used in conjunction with negative mode ionization to provide fragment ions of certain glycan structural features (11, 14). The glycopeptide-based approach allowed analysis of CD52 glycans directly bound to the peptide backbone with the assurance of no interference by Fc glycan. The two approaches largely corroborated each other, adding confidence in the reported structures. Indeed, we found the same results after CD52-Fc fractionation by anion exchange chromatography, as described. Anion exchange was previously employed to fractionate sialylated glycoforms of the soluble and sperm-associated form of CD52 in the mouse reproductive tract (16), but glycan structure was not analyzed.

We confirmed the importance of the α-2,3 sialic acid linkage for CD52-Fc bioactivity. Previously, we showed that soluble CD52 mediates T-cell suppression by binding to Siglec-10 (3). The diverse family of mostly inhibitory Siglec receptors has evolved to recognize linkage-specific sialic acid residues on host cells and pathogens (17). Siglec-10 is highly expressed on leukocytes (18, 19) and plays significant roles in regulating the innate and adaptive immune response to tissue injury, sepsis and viral invasion (20). Previously, Siglec-10 was reported to have no binding preference for α-2,3 or α-2,6-sialylation (18, 21). However, we recently found that human CD52-Fc binds to Siglec-10 preferentially through the α-2,3 sialic acid linkage (4). In the present study, bioactive CD52-Fc was characterized by a high abundance of the α-2,3 sialic acid linkage, and re-sialylation with α-2,3 restored the bioactivity of sialidase-treated CD52-Fc.

Regarding CD52 *O-*glycosylation, Ermini et al. (22) deduced the presence of *O-*glycosylation of CD52 by antibody binding, but did not determine the type, occupancy or localization of *O-*glycans. We characterized for the first time the *O*-glycans on human spleen CD52. In addition, recombinant CD52-Fc was found to contain a low abundance (4%) of mainly core type 2 *O*-glycans with one or two sialic acid residues, on Ser 12 and Thr 8, but this increased significantly (to 15–20%) in MonoQ-purified bioactive CD52-Fc. Due to the proximity of the *N-* and *O-*glycosylation sites of CD52 peptide, the low degree of *O*-glycosylation could be due to steric hindrance from the bulky *N-*glycan. Determination of the *O-*glycan sites and occupancies on human spleen CD52 was challenging due to its limited availability. However, with continuing developments in highly sensitive glycoprotemics (20) it should soon be possible to identify the site-specific *O-*glycosylation of CD52 directly from tissues and bodily fluids without prior purification. Our results also indicate that recombinant human CD52 does not require fucosylated *O*-glycans for bioactivity, as found for CD52 of the male reproductive tract (23). The polypeptide of recombinant human CD52 is identical to human spleen CD52 and shares the core type 2 and core type 2 sialylated *O*-glycans with reproductive tract CD52 (24). However, we identified a dramatic enrichment of *O-*glycosylation in the MonoQ active CD52-Fc fractions, strongly implying a role for both *N*- and *O-*glycosylation in the bioactivity of CD52.

Another striking observation was the inverse association between CD52 bioactivity and bisecting GlcNAcylation. Previously, *N*-glycans displaying bisecting GlcNAc were found to correlate with a decrease in tri- and tetra-sialylated structures, since bisecting GlcNAc residues inhibit the activity of GlcNAc-transferases required to generate multi-antennary sialoglycans (25). Furthermore, an increase in bisecting GlcNAcylation has been linked with a decrease in α-2,3 sialylation (26), which we here show is important for CD52 bioactivity. The functions of bisecting GlcNAc are not fully understood, but they have been associated with a decrease in target-cell susceptibility for NK cell-induced lysis (27). Interestingly, CD52 in recombinant human CD52-Fc resembled naturally-occurring CD52 purified from human spleen with respect to *N-* and *O-*glycosylation, except in the degree of polyLacNAc elongation, which was greater in the native form. Although bioactive CD52 was characterized by higher abundance of sialylated structures and polyLacNAcs, the contribution of polyLacNAc units to CD52 activity is yet to be determined.

In conclusion, the comparison of native and recombinant human CD52-Fc, and CD52-Fc variants differing in bioactivity, enabled us to identify glycoform features that underlie the immune suppressive activity of CD52. These can be summarized as an abundance of tri- and tetra-antennary α-2,3-sialylated *N*-glycans, an absence of bisecting GlcNAcylation and the presence of the di-sialylated type 2 *O-*glycosylation. Further glycomic analysis will be required to detail the length of polyLacNAc extensions and the degree of polyLacNAc branching. The present study extends our knowledge of the glycan structure required for CD52 bioactivity and may assist in the design and production of CD52-Fc as an immunotherapeutic agent.

## Ethics Statement

Cells were isolated from human blood buffy coats (Australian Red Cross Blood Service, Melbourne, VIC, Australia) or blood of de-identified healthy volunteers with informed consent through the Volunteer Blood Donor Registry of The Walter and Eliza Hall Institute of Medical Research (WEHI), following approval by WEHI and Melbourne Health Human Ethics Committees. Healthy human spleen from cadaveric organ donors were obtained from Australian Islet Transplant Consortium and trained coordinators of Donate Life from heart-beating, brain dead donors with informed written consent of next of kin. All studies were approved by WEHI Human Research Ethics Committee (Project 05/12).

## Author Contributions

LH initiated the study and all authors contributed to its design. EB-S, AS, and AJ performed most of the experiments. AS, EB-S, MT-A, AE-D, NP, and LH analyzed data and drafted the manuscript. EG-B and TA provided advice and technical support. All authors discussed and commented on the manuscript.

### Conflict of Interest Statement

The authors declare that the research was conducted in the absence of any commercial or financial relationships that could be construed as a potential conflict of interest.

## Supplementary Material

Supplementary Figure 1 Analysis of CD52 I and CD52 II at the intact peptide level. **(A)** The theoretical isotopic distribution of deconvoluted 5871.99 (amu) CD52 glycoform as tri-sialylated (GlcNAc_5_Man_3_Gal_3_NeuAc_3_Fucose_1_) or di-sialylated with two outer fucoses (GlcNAc_5_Man_3_Gal_3_NeuAc_2_Fucose_3_). The bar graph shows the theoretical isotopic envelopes generated when different amount of these two glycans are present. Experimental isotopic distribution values suggest a population of 90–100% tri-sialylated structures. **(B)** High-resolution intact mass analysis of CD52 I (pink) and CD52 II (green).

Supplementary Figure 2 CD52-Fc III fractions resolved in isoelectric focusing (IEF) gel. Colloidal Coomassie Blue gel showing protein in MonoQ fractions (F29–54). Fractions showed a gradual decrease in isoelectric point (pI) values.

Supplementary Figure 3 High-resolution intact mass analysis of MonoQ fractions (F30 and F47–50). **(A)** F30 intact mass analysis of the CD52 III part showed absence of sialic acid molecules. **(B)** MonoQ fractionation was able to separate CD52 sialylated structures according to their amount of sialic acid as well as number of antennae. Among fractions F47–50, F49, and F50 contained more of the bigger sialylated structures.

Supplementary Figure 4 Active MonoQ fractions suppress in a dose-dependent manner. **(A,B)** IFN-γ production measured by ELISpot assay from human PBMCs (2 × 10^5^) incubated in IP5 medium with no antigen or anti-CD3/CD28 antibody Dynabeads. **(A)** Active Mono-Q fractions (F48–49) suppressed in a dose-dependent manner (0.3125, 0.625, 1.25, 2.5, and 5 μg/ml). **(B)** Adjacent fractions (inactive; F46, F47, F50, and F51) do not suppress despite the increase of protein added (5, 10, 20, and 40 μg/ml). The data points in panels **(A,B)** are plotted as mean ± SEM of three independent replicates.
