# Supplementary figures and images for: Specific Sialoforms Required for the Immune Suppressive Activity of Human Soluble CD52
Source: Front Immunol. 2019 Aug 27;10:1967. doi: 10.3389/fimmu.2019.01967 (PMC6719568; doi:10.3389/fimmu.2019.01967)

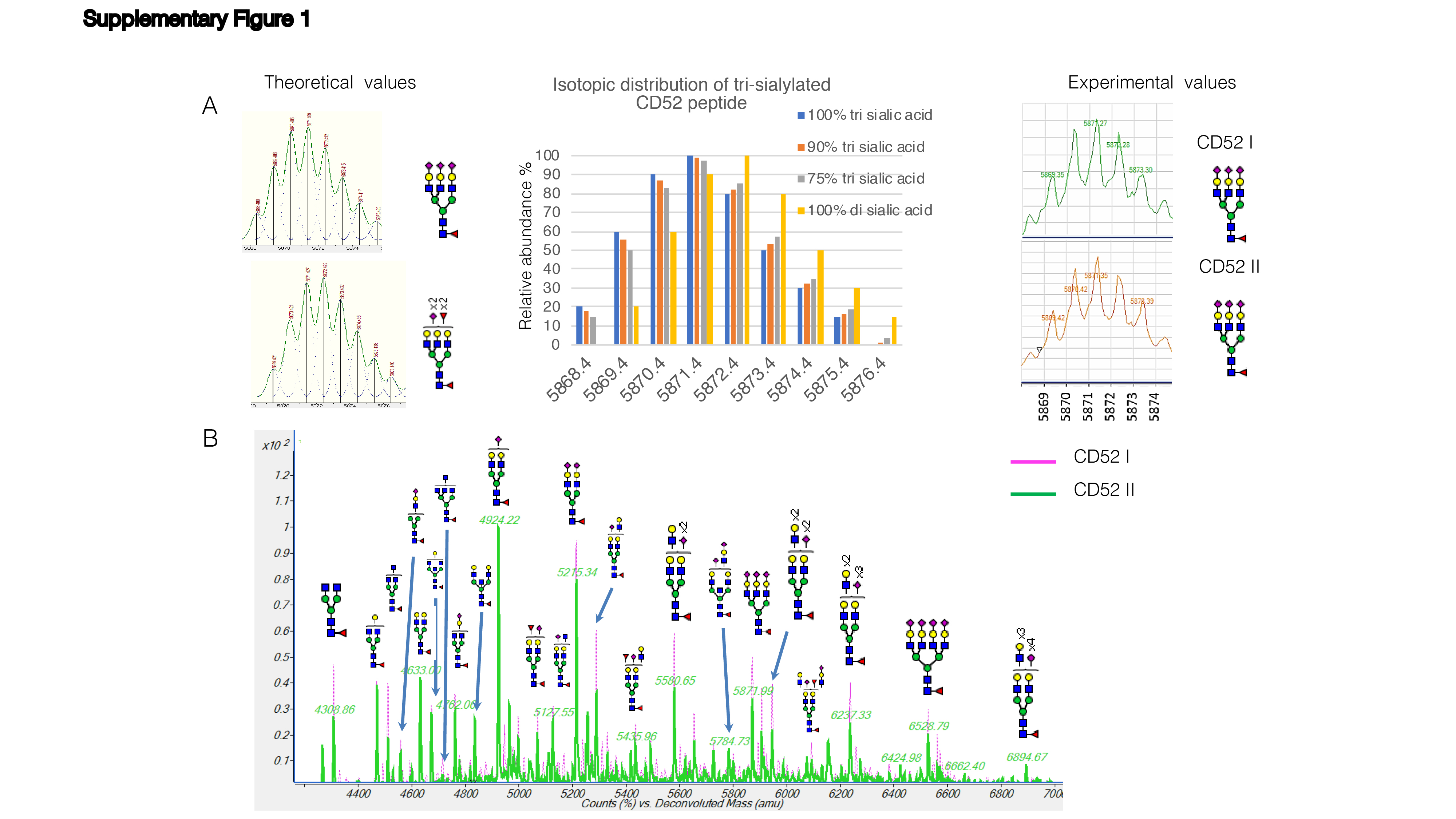

Supplement: Supplementary Figure 1 — Analysis of CD52 I and CD52 II at the intact peptide level. (A) The theoretical isotopic distribution of deconvoluted 5871.99 (amu) CD52 glycoform as tri-sialylated (GlcNAc5Man3Gal3NeuAc3Fucose1) or di-sialylated with two outer fucoses (GlcNAc5Man3Gal3NeuAc2Fucose3). The bar graph shows the theoretical isotopic envelopes generated when different amount of these two glycans are present. Experimental isotopic distribution values suggest a population of 90–100% tri-sialylated structures. (B) High-resolution intact mass analysis of CD52 I (pink) and CD52 II (green). [file Image_1.jpg]

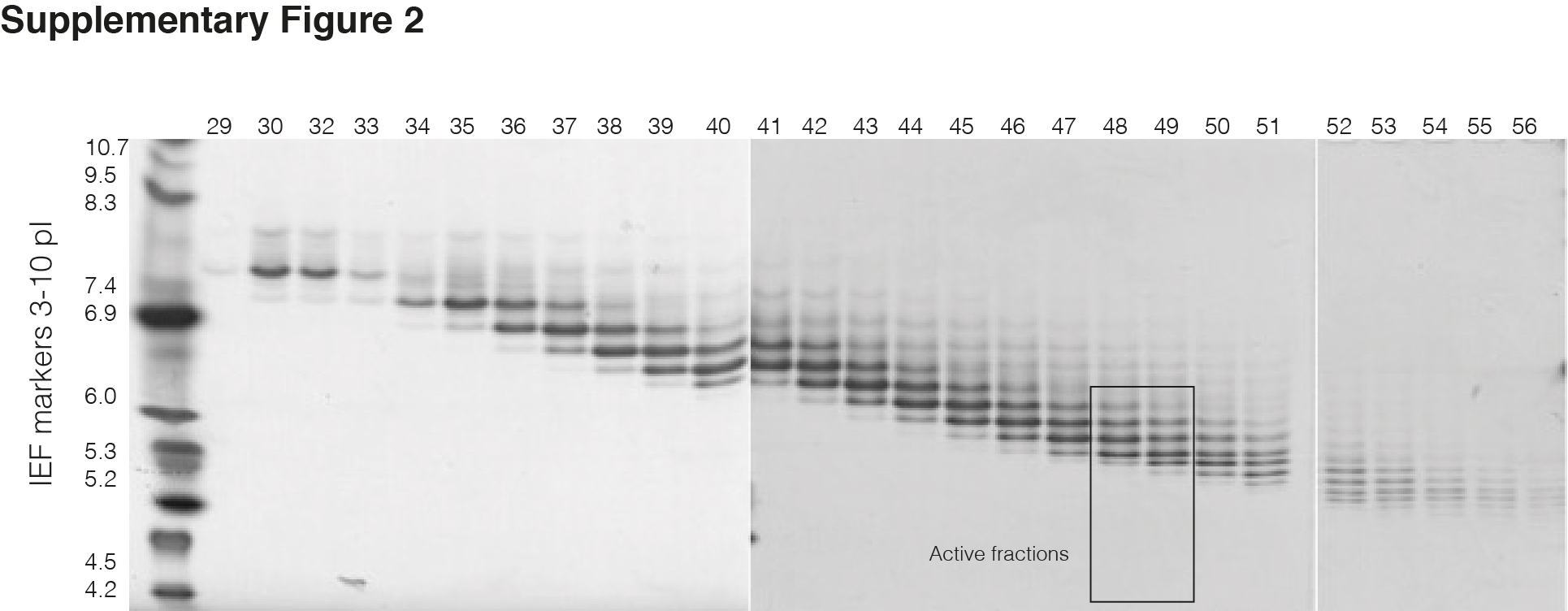

Supplement: Supplementary Figure 2 — CD52-Fc III fractions resolved in isoelectric focusing (IEF) gel. Colloidal Coomassie Blue gel showing protein in MonoQ fractions (F29–54). Fractions showed a gradual decrease in isoelectric point (pI) values. [file Image_2.jpg]

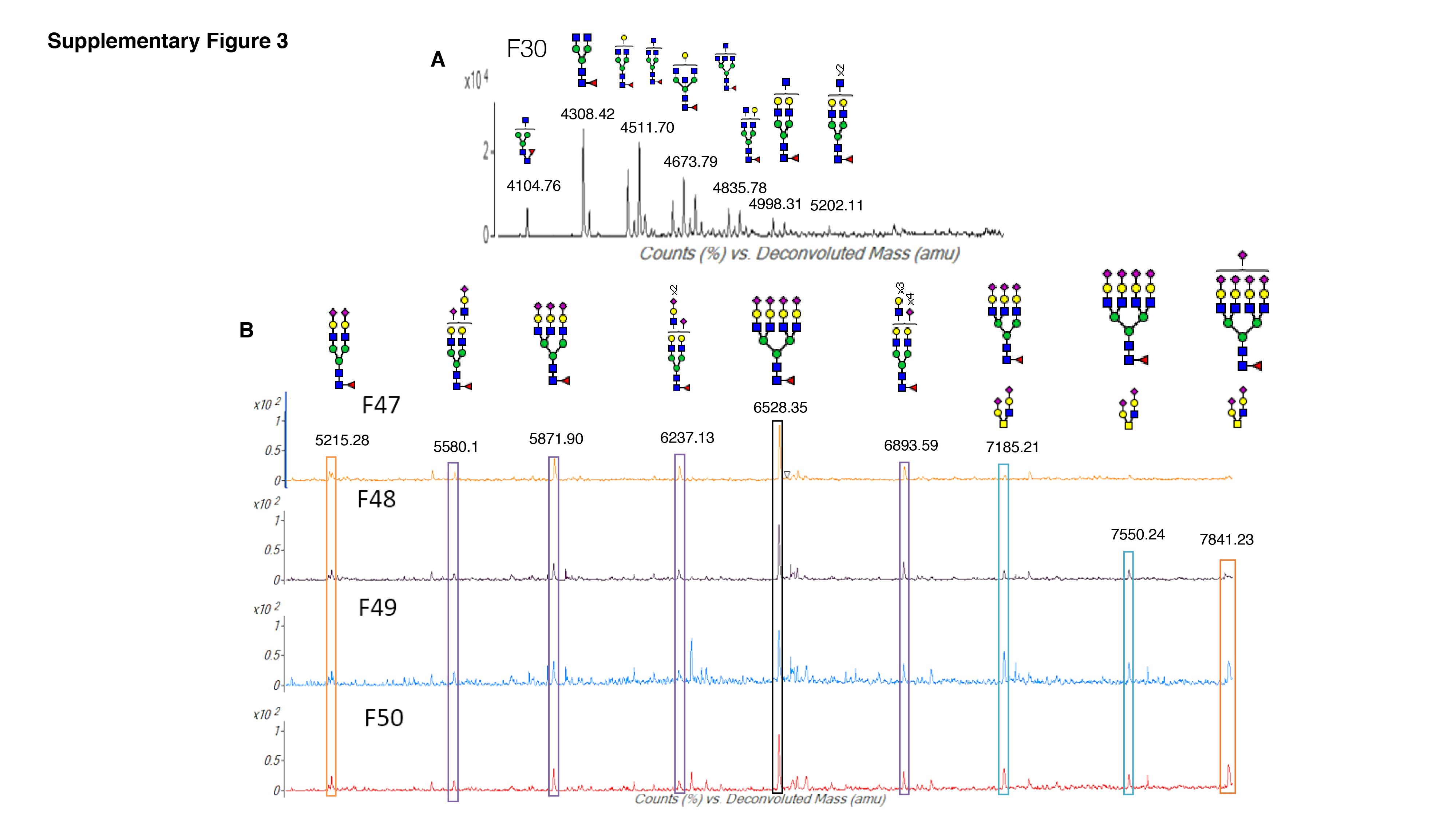

Supplement: Supplementary Figure 3 — High-resolution intact mass analysis of MonoQ fractions (F30 and F47–50). (A) F30 intact mass analysis of the CD52 III part showed absence of sialic acid molecules. (B) MonoQ fractionation was able to separate CD52 sialylated structures according to their amount of sialic acid as well as number of antennae. Among fractions F47–50, F49, and F50 contained more of the bigger sialylated structures. [file Image_3.jpg]

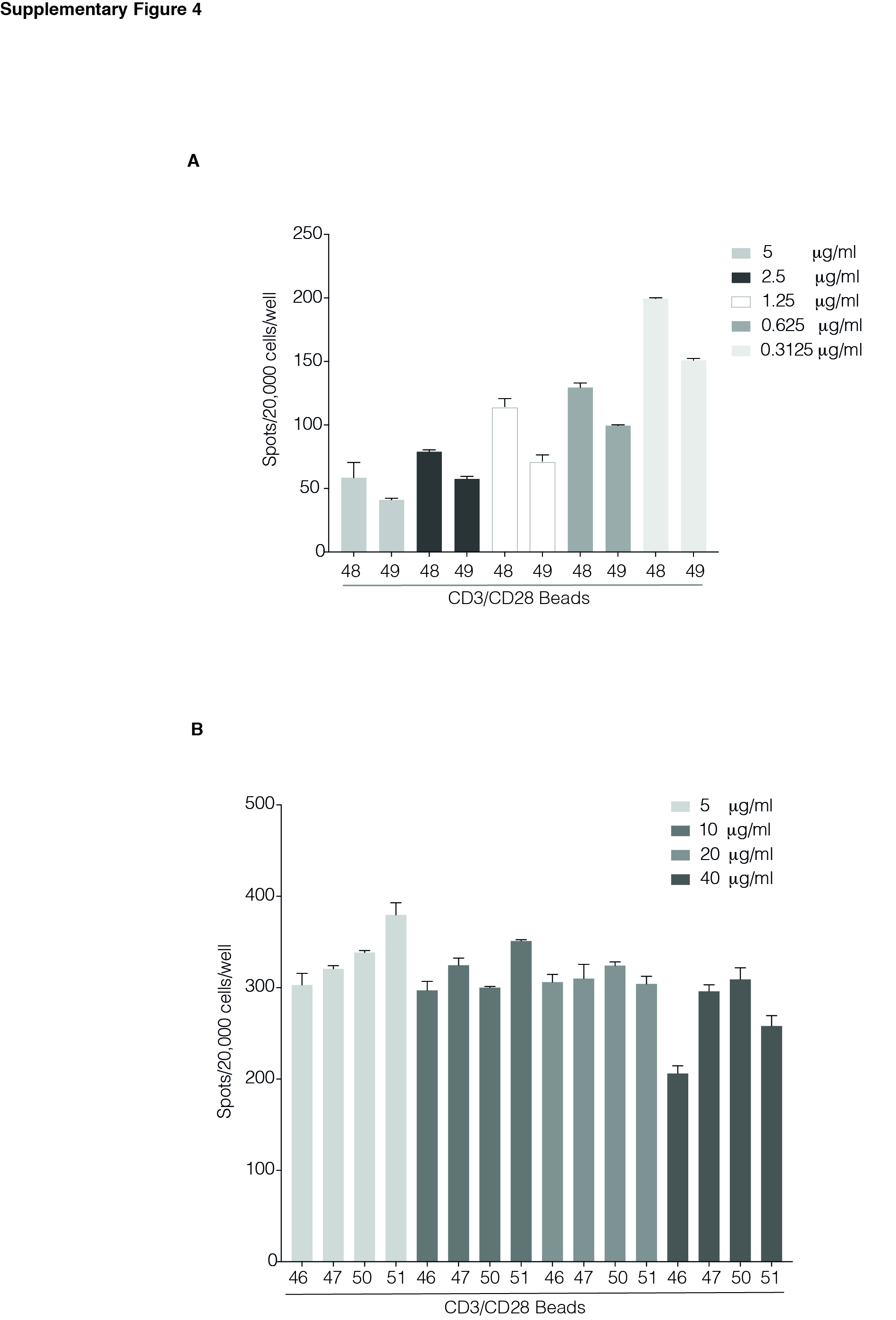

Supplement: Supplementary Figure 4 — Active MonoQ fractions suppress in a dose-dependent manner. (A,B) IFN-γ production measured by ELISpot assay from human PBMCs (2 × 10^5^) incubated in IP5 medium with no antigen or anti-CD3/CD28 antibody Dynabeads. (A) Active Mono-Q fractions (F48–49) suppressed in a dose-dependent manner (0.3125, 0.625, 1.25, 2.5, and 5 μg/ml). (B) Adjacent fractions (inactive; F46, F47, F50, and F51) do not suppress despite the increase of protein added (5, 10, 20, and 40 μg/ml). The data points in panels (A,B) are plotted as mean ± SEM of three independent replicates. [file Image_4.jpg]
